# Increasing maternal age associates with lower placental *CPT1B* mRNA expression and acylcarnitines, particularly in overweight women

**DOI:** 10.3389/fphys.2023.1166827

**Published:** 2023-05-18

**Authors:** Hannah E. J. Yong, Oliver C. Watkins, Tania K. L. Mah, Victoria K. B. Cracknell-Hazra, Reshma Appukuttan Pillai, Preben Selvam, Mohammad O. Islam, Neha Sharma, Amaury Cazenave-Gassiot, Anne K. Bendt, Markus R. Wenk, Keith M. Godfrey, Rohan M. Lewis, Shiao-Yng Chan

**Affiliations:** ^1^ Singapore Institute for Clinical Sciences (SICS), Agency for Science, Technology and Research (A*STAR), Singapore, Singapore; ^2^ Department of Obstetrics and Gynaecology, Yong Loo Lin School of Medicine, National University of Singapore, Singapore, Singapore; ^3^ NIHR Southampton Biomedical Research Centre, University of Southampton and University Hospital Southampton NHS Foundation Trust, Southampton, United Kingdom; ^4^ Department of Biochemistry and Precision Medicine Translational Research Programme, Yong Loo Lin School of Medicine, National University of Singapore, Singapore, Singapore; ^5^ Singapore Lipidomics Incubator, Life Sciences Institute, National University of Singapore, Singapore, Singapore; ^6^ MRC Lifecourse Epidemiology Centre, University of Southampton, Southampton, United Kingdom; ^7^ Institute of Developmental Sciences, Faculty of Medicine, University of Southampton, Southampton, United Kingdom

**Keywords:** placenta, maternal age, lipid metabolism, carnitine palmitoyltransferases, obesity, overweight, CPT1B

## Abstract

Older pregnant women have increased risks of complications including gestational diabetes and stillbirth. Carnitine palmitoyl transferase (CPT) expression declines with age in several tissues and is linked with poorer metabolic health. Mitochondrial CPTs catalyze acylcarnitine synthesis, which facilitates fatty acid oxidization as fuel. We hypothesized that the placenta, containing maternally-inherited mitochondria, shows an age-related CPT decline that lowers placental acylcarnitine synthesis, increasing vulnerability to pregnancy complications. We assessed *CPT1A*, *CPT1B*, *CPT1C* and *CPT2* mRNA expression by qPCR in 77 placentas and quantified 10 medium and long-chain acylcarnitines by LC-MS/MS in a subset of 50 placentas. Older maternal age associated with lower expression of placental *CPT1B*, but not *CPT1A*, *CPT1C* or *CPT2*. *CPT1B* expression positively associated with eight acylcarnitines and *CPT1C* with three acylcarnitines, *CPT1A* negatively associated with nine acylcarnitines, while *CPT2* did not associate with any acylcarnitine. Older maternal age associated with reductions in five acylcarnitines, only in those with BMI≥ 25 kg/m^2^, and not after adjusting for *CPT1B* expression. Our findings suggest that *CPT1B* is the main transferase for placental long-chain acylcarnitine synthesis, and age-related *CPT1B* decline may underlie decreased placental metabolic flexibility, potentially contributing to pregnancy complications in older women, particularly if they are overweight.

## 1 Introduction

More women are entering pregnancy at an older age worldwide, particularly in developed countries. These women are at increased risk of pregnancy complications including gestational diabetes, pre-eclampsia and stillbirth (Flenady et al., 2011; Lean et al., 2017; Saccone et al., 2022). Nonetheless, the mechanistic pathways by which older age contributes to adverse pregnancy outcomes are still unclear (Huang et al., 2008; Plows et al., 2018). It is thus important to examine potential mechanisms by which advanced maternal age might affect pregnancy outcomes to aid development of strategies to reduce risk.

Outside of pregnancy, several studies have reported that expression and activity of carnitine palmitoyltransferases (CPTs) decline with age in multiple tissues and that these changes associate with poorer metabolic health. Aging is associated with decreased CPT1 activity in rodent hearts (McMillin et al., 1993; Odiet et al., 1995), where the predominant CPT1 isoform is CPT1B. In mice, genetically-induced deficiency or age-associated reduction of skeletal muscle CPT1B expression leads to the development of insulin resistance provoked by a high fat diet challenge (Kim et al., 2014; Vieira-Lara et al., 2021). Such relationships are consistent with a study in elderly humans showing that higher skeletal *CPT1B* mRNA expression associated with insulin sensitivity and better metabolic health (Bétry et al., 2019). A negative association of CPT1 expression with age is also observed in peripheral blood mononuclear cells (Karlic et al., 2003).

CPTs catalyze the synthesis of acylcarnitines from fatty-acyl CoAs, a process essential to facilitate the transport of fatty acids into mitochondria for fatty acid oxidation (also known as beta oxidation), and for the production of acylcarnitines for cellular use, secretion and signaling (Ceccarelli et al., 2011; Houten et al., 2020). CPTs are expressed in most tissues with the ratios of isoforms dependent on the tissue type and species (Ceccarelli et al., 2011; Houten et al., 2020). CPT1 and CPT2 are present on the outer and inner mitochondrial membrane respectively and together enable fatty acids to be transported across the mitochondrial membrane as acylcarnitines for utilization (Ceccarelli et al., 2011; Houten et al., 2020). CPT1 is the main regulator of fatty acid oxidation and occurs as three isoforms—CPT1A, CPT1B and CPT1C; their individual characteristics remain under investigation (Ceccarelli et al., 2011; Virmani et al., 2015; Houten et al., 2020). CPTs are also active in peroxisomes and the endoplasmic reticulum, but their role in these organelles is not well understood (Sierra et al., 2008; Ceccarelli et al., 2011; Houten et al., 2020).

In the placenta, CPTs are important for generating acylcarnitines, for use locally as well as for release into both fetal and maternal circulations to serve as both a fuel source and a precursor of activated fatty acids for lipid remodeling and protein palmitoylation (Novak et al., 1981; Schmidt-Sommerfeld et al., 1985; Arduini et al., 1992; Arduini et al., 1994; Lahjouji et al., 2004; Jones et al., 2010). These exported acylcarnitines act as signaling molecules, anti-oxidants and as an alternative fetal fuel source to glucose (Jones et al., 2010; Yli and Kjellmer, 2016; Kolb et al., 2021). Hence, acylcarnitine supply is vital to the fetus when glucose and oxygen supply is limited and when anaerobic metabolism and oxidative stress is high such as during parturition (Jones et al., 2010; Yli and Kjellmer, 2016; Kolb et al., 2021). Indeed, increased umbilical cord blood acylcarnitines are associated with both extremes of birthweight (Giannacopoulou et al., 1998; Sánchez-Pintos et al., 2016; El-Wahed et al., 2017; Sánchez-Pintos et al., 2017), where there is often either a lack or oversupply of nutrients relative to fetal needs.

Therefore, given that developmentally, mitochondria in conceptuses and, hence, placental mitochondrial CPTs are maternally-inherited, we hypothesized that a maternal age-related decline in placental CPT expression and activity may contribute to the development of pregnancy adversity. As an initial step, our study aimed to determine the relationship between maternal age and the placental expression of four CPT isoforms, and associated alterations in placental acylcarnitine abundance.

## 2 Materials and methods

### 2.1 Subject recruitment and placental collection

Placentas were collected at term elective cesarean sections of singleton pregnancies at the National University Hospital, Singapore with written informed consent. Only elective cesarean section cases were included to reduce the possible effects of labor on placental expression of CPTs and acylcarnitine content. Indications for elective cesarean section were previous cesarean section, breech presentation, suspected macrosomia or maternal request/social reasons. Participants were of Asian ethnicity (classified as Chinese and non-Chinese: Malay or Indian), self-reported non-smokers, conceived spontaneously and delivered neonates that were not small-for-gestational age (birthweight >10th centile). All participants underwent a routine 75 g oral glucose tolerance test (OGTT) after an overnight fast during pregnancy. Gestational diabetes mellitus (GDM) was diagnosed according to World Health Organization 2013 criteria of a fasting glucose 5.1—6.9 mmol/L, and/or 1 h glucose ≥10.0 mmol/L, and/or 2 h glucose 8.5—11.0 mmol/L (World Health Organization Guideline, 2014). With the exception of GDM in 39 subjects, all participants were otherwise healthy and had uncomplicated pregnancies (Table 1). Ethics approval was granted by the National Healthcare Group Domain Specific Research Board (References 2000/00524 and 2016/00183).

**TABLE 1:** Participant characteristics by molecular analysis method.

Characteristics^a^	RT-qPCR analysis (n = 77)	Lipidomic analysis (n = 50)
Maternal age (years)	33.1 ± 3.5	32.8 ± 3.8
Maternal ethnicity (n)	45 Chinese, 32 non-Chinese	27 Chinese, 23 non-Chinese
Maternal BMI in early pregnancy (kg/m^2^)	25.5 ± 5.1	25.7 ± 5.1
Gestational diabetes mellitus status (n, %)	39 (50.6%)	24 (48.0%)
Antenatal fasting glycemia (mmol/L)^b^	4.5 ± 0.3	4.5 ± 0.4
Antenatal 2h glycemia (mmol/L)^b^	7.4 ± 1.8	7.3 ± 2.0
Gestational age at delivery (weeks)	38.6 ± 0.6	38.5 ± 0.5
Infant sex (n males, n females)	44 Males, 33 Females	30 Males, 20 Females
Infant birthweight (g)	3276.9 ± 313.3	3310.8 ± 297.3
Customized infant birthweight percentiles (%)^c^	56.8 ± 25.5	60.2 ± 23.4

^a^ Data presented as mean ± standard deviation unless stated otherwise.

^b^ Subjects underwent a 75 g oral glucose tolerance test conducted during pregnancy.

^c^ Customized for maternal BMI, ethnicity, parity, gestational age at delivery and infant sex.

### 2.2 Sample processing

Five villous tissue biopsies were obtained from random sampling across each placenta. Following removal of the maternal decidua, biopsies were snap frozen in liquid nitrogen within 10 min of delivery and stored at −80°C until use. Considering variation across each placenta, biopsies for each placenta were subsequently pulverized in liquid nitrogen and mixed together for RNA and lipid extractions.

### 2.3 RNA extraction, cDNA synthesis and real-time quantitative polymerase chain reaction (RT-qPCR)

Placental mRNA expression of carnitine palmitoyltransferases was determined as described previously (Watkins et al., 2022). Briefly, following phenol-chloroform extraction, placental RNA was purified with the RNeasy Mini Kit (Qiagen) and reverse transcribed to cDNA with Superscript III reverse transcriptase (Thermo Fisher Scientific) according to manufacturer’s instructions. RT-qPCR was performed with TaqMan Fast Advanced Master Mix (Thermo Fisher Scientific) on an Applied Biosystems 7500 Fast Real-Time PCR System (Thermo Fisher Scientific). Samples were run in duplicate 10 µL reactions containing 5 ng cDNA at the following settings: 95°C for 20 s, followed by 45 cycles of 95°C for 3 s and 60°C for 30 s. Inventoried FAM-labeled TaqMan probes were used for three housekeeping genes—*CYC1* (cytochrome C1, Hs00357718_m1), *SDHA* (succinate dehydrogenase complex, subunit A, Hs00188166_m1) and *TBP* (TATA-box binding protein, Hs00427620_m1); and four CPT family genes—*CPT1A* (Hs00912671_m1), *CPT1B* (Hs00993896_g1), *CPT1C* (Hs00380581_m1) and *CPT2* (Hs00988962_m1). The average threshold cycle (C_T_) value of non-GDM subjects served as the calibrator (assigned value of 1) for relative quantification. Relative expression of each CPT isoform was calculated using formula 2^(−ΔCT)^ and normalized to the geometric mean expression of the three housekeeping genes.

### 2.4 Lipid extraction and quantification by liquid chromatography tandem mass spectrometry (LC-MS/MS)

Lipid extraction and quantification by LC-MS/MS was performed on a subset of 50 placentas using methods similar to previous work on the placental lipidome (Wong et al., 2021; Watkins et al., 2022). In brief, approximately 250 mg of each placental sample was freeze-dried, weighed and homogenized in 1 ml phosphate buffered saline. Following an addition of 800 µL butanol/methanol (1:1) to 40 µL of placental homogenate and 10 µL of internal standard mix containing 151.1 pmol acylcarnitine 16:0 d3 (Larodan Chemicals, Solna, Sweden), samples were vortexed briefly, sonicated for 30 min in an ice bath and shaken for 30 min at 4°C. After centrifugation at 13,000 rpm for 10 min, the supernatant was collected into a La-Pha-Pack HPLC tube (Langerwehe, Germany) and stored at -80°C until LC-MS/MS analysis. Quality control (QC) samples were similarly prepared from placental homogenates pooled from several subjects. Lipid extracts (5 µL) were then injected into an Agilent 6490 triple quadrupole LC-MS/MS instrument with chromatography performed as described in Supplementary Methods. Metabolite peak areas were integrated using Mass Hunter QQQ Quantitative Analysis Version 10. Lipids were considered quantifiable if their %RSD in QC samples was less than 25% and the peak area at least 10 times that of a blank sample extracted under the same conditions. Placental lipid content was expressed as µmol lipid/mg tissue dry weight. Ten medium and long chain acylcarnitines (12:0, 14:0, 14:1, 14:2, 15:0, 16:0, 16:1, 18:0, 18:1 and 18:2) were measured.

### 2.5 Statistical analysis

To ensure a normal distribution and to standardize comparisons between genes and lipids that had different degrees of interindividual variability, gene expression and lipid data were log2-transformed and then converted to Z-scores for analysis. Linear regression models were run in R version “Kick Things” with ‘tidyverse’ Version: 1.3.1 (Wickham et al., 2019). To account for multiple testing and minimize false discovery, Benjamini–Hochberg correction was applied with statistical significance set at *p* < 0.05. For each CPT, linear regression was first performed between gene expression (outcome) and maternal age. This model was then rerun with covariate adjustment for maternal fasting glycemia, ethnicity, maternal BMI, gestational age and infant sex. To determine how CPT expression might influence the production of placental acylcarnitines, linear regression was performed between placental acylcarnitine abundance (outcome) and gene expression (for each CPT). Lastly, to explore if maternal factors such as high maternal glycemia and BMI affected the relationship between placental CPT expression with maternal age and acylcarnitine abundance, these associations were examined in the study population as a whole, as well as following stratification by GDM or BMI status, as decided *a priori*.

## 3 Results

### 3.1 Clinical characteristics of study participants

Study participants (n = 77) for the RT-qPCR analysis were predominantly of Chinese ethnicity, with a mean age of 33 years, a mean BMI in early pregnancy of 25.5 kg/m^2^ and delivered at an average of 38.6 weeks of gestation (Table 1). Approximately 50% of these women had GDM, with an average antenatal OGTT fasting and 2 h glucose of 4.5 and 7.4 mmol/L respectively. The maternal characteristics of the subset (n = 50) used for the lipidomic analysis were similar to those used for the RT-qPCR analysis. The proportion of male to female infants, birthweight mean and average birthweight centiles were also comparable between both groups.

### 3.2 Participant characteristics associated with placental expression of CPT isoforms

**FIGURE 1:**
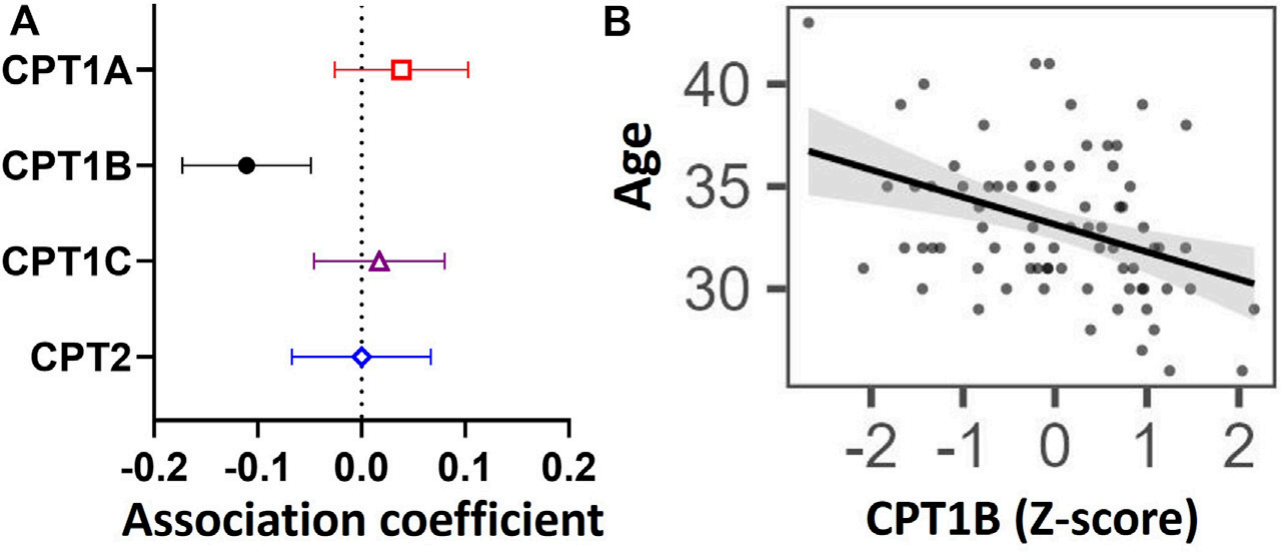
Associations between placental mRNA expression of CPT isoforms and maternal age in n = 77 placentas. Forest plot **(A)** shows coefficient estimates and 95% confidence intervals of associations between each CPT isoform (outcome) and age (years), after adjusting for maternal fasting glycemia, ethnicity, maternal BMI, gestational age and fetal sex. Scatter plot **(B)** shows the unadjusted relationship between maternal age and Z-score for *CPT1B* mRNA expression. A black line denotes a significant association. The shaded grey area represents the 95% confidence interval of the regression. Placental mRNA expression data of CPTs were log_2_-transformed then converted to Z-scores prior to linear regression.

Older maternal age was associated with lower placental *CPT1B* expression [coefficient estimate: -0.107 (-0.167, -0.047) Z-score of expression per year, *p* = 0.001], but not with that of *CPT1A* [0.043 (-0.021, 0.108), *p* > 0.05], *CPT1C* [0.018 (-0.047, 0.083), *p* > 0.05] or *CPT2* [0.003 (-0.062, 0.068), *p* > 0.05] (Figure 1). Associations remained similar [*CPT1B* coefficient estimate: -0.111 (-0.173, -0.049), *p* = 0.006] after covariate adjustment for maternal fasting glycemia, ethnicity, maternal BMI, gestational age and infant sex. No associations were observed between maternal fasting glycemia, ethnicity, maternal BMI, gestational age or fetal sex and the expression of any CPT isoform.

### 3.3 Association of expression of CPT isoforms with acylcarnitines in the placenta

**FIGURE 2:**
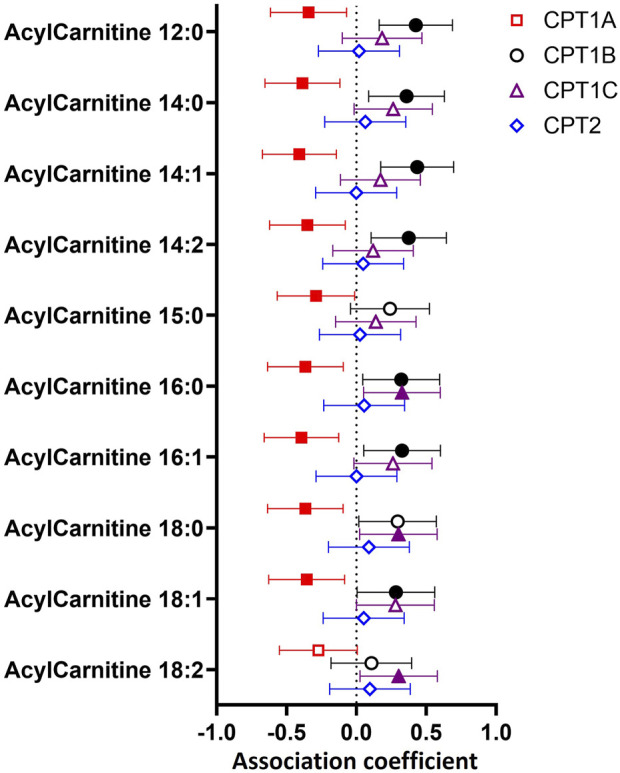
Associations between mRNA expression of CPT isoforms and placental acylcarnitines by linear regression. The forest plot shows coefficient estimates and 95% confidence intervals of associations between CPT isoforms and placental acylcarnitines (outcome, n = 50 placentas). Filled symbols show acylcarnitines that are significantly associated after adjustment by Benjamini–Hochberg’s correction. Data for placental acylcarnitine abundance and mRNA expression data of CPT isoforms were log2-transformed then converted to Z-scores prior to linear regression.

To determine whether variations in CPT isoform expression related to differences in transferase activity represented by placental acylcarnitine content, we examined the relationship between expression of each CPT isoform with 10 medium and long-chain acylcarnitines in the placenta (Figure 2). Placental *CPT1A* expression negatively associated with nine acylcarnitines (12:0, 14:0, 14:1, 14:2, 15:0, 16.0, 16:1, 18:0, 18:1), while *CPT1B* positively associated with eight acylcarnitines (12:0, 14:0, 14:1, 14:2, 16.0, 16:1, 18:0, 18:1) and *CPT1C* positively associated with three long chain acylcarnitines (16:0, 18:0 and 18:2). The exception was *CPT2,* which showed no associations with any acylcarnitine.

### 3.4 Associations between *CPT1B* expression and maternal age with placental acylcarnitines following stratification by maternal BMI or GDM status

**FIGURE 3:**
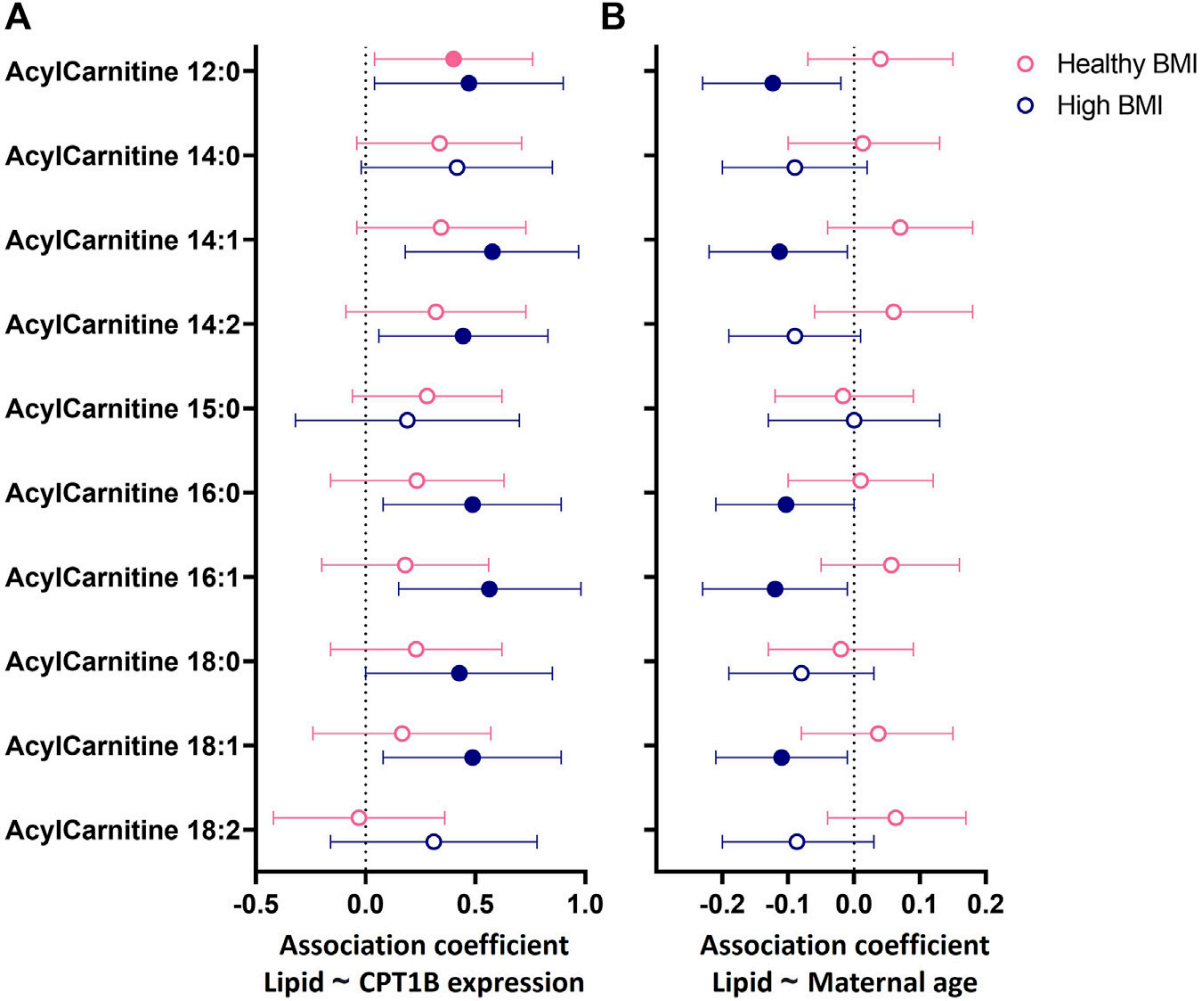
Associations between **(A)** placental *CPT1B* mRNA expression or **(B)** maternal age with the abundance of placental acylcarnitines by linear regression stratified by maternal BMI status. The forest plots show coefficient estimates and 95% confidence intervals of associations between placental *CPT1B* mRNA expression **(A)** or maternal age (years, **(B)** with placental acylcarnitines (outcome) in subjects with healthy (<25 kg/m^2^, n = 25) and high (≥25 kg/m^2^, n = 25) BMI. Filled symbols show acylcarnitines that are significantly associated after adjustment by Benjamini–Hochberg’s correction. Data for placental acylcarnitine abundance and mRNA expression data of *CPT1B* were log_2_-transformed then converted to Z-scores prior to linear regression.

Both a high maternal BMI (≥25 kg/m^2^) and GDM are known to increase the supply and availability of fatty acids to the placenta (Duttaroy and Basak, 2021), which could place greater stress on the placenta’s capacity for fatty acid processing. Thus, to determine whether such factors altered the relationships of *CPT1B* expression and maternal age with placental acylcarnitines, we examined these associations following stratification by BMI (Figure 3) and GDM status (Supplementary Figure S1).

In BMI-stratified analyses (Figure 3), among participants with a healthy BMI (<25 kg/m^2^; n = 25), *CPT1B* expression positively associated with only one acylcarnitine (12:0). In contrast, in those with a high BMI (≥25 kg/m^2^; n = 25), *CPT1B* expression remained strongly positively associated with seven acylcarnitines (12:0, 14:1, 14:2, 16.0, 16:1, 18:0, 18:1). Only acylcarnitine 12:0 was significantly associated with *CPT1B* expression with similar coefficient estimates in both BMI groups. Differences by GDM status were less apparent, with the normoglycemic group showing significant positive associations for two acylcarnitines (12:0, 14:1) and the GDM group for three acylcarnitines (14:1, 16:0, 16:1), with an overlap observed for acylcarnitine 14:1 (Supplementary Figure S1A).

Since placental *CPT1B* expression declined with older maternal age, it was expected that placental acylcarnitines would be negatively associated with age. However, no direct relationships between maternal age and placental acylcarnitines were observed (Supplementary Table S3). Instead, we only observed such a relationship of older maternal age with lower acylcarnitines (12:0, 14:1, 16:0, 16:1, 18:1) in those with a high BMI and not in those with a healthy BMI (Figure 3B). No relationships were seen when stratified by GDM status (Supplementary Figure S1B). The associations seen for the high BMI group remained similar after adjusting for maternal ethnicity, fasting glycemia, gestational age at delivery and infant sex. Following adjustment for placental *CPT1B* expression, all associations between maternal age and placental acylcarnitines were attenuated and no longer significant.

## 4 Discussion

### 4.1 Main findings

Our study demonstrates that older maternal age is associated with lower placental expression of *CPT1B*, but not that of *CPT1A*, *CPT1C* or *CPT2*. Furthermore, placental *CPT1B* expression positively associated with eight out of 10 acylcarnitines quantified in the placenta, suggesting it may play a prominent role in placental long-chain acylcarnitine synthesis. Placental acylcarnitines were only reduced with older maternal age in overweight/obese participants. These associations between maternal age and placental acylcarnitines were attenuated after accounting for *CPT1B* expression.

### 4.2 Implications of reduced CPT1B expression in the placenta

The inverse relationship between maternal age and placental *CPT1B* mRNA expression is consistent with past studies in elderly humans and aged rodents demonstrating decreased expression and activity of CPT in tissues such as the heart and skeletal muscles, where the CPT1B is the predominant isoform (McMillin et al., 1993; Odiet et al., 1995; Bétry et al., 2019; Vieira-Lara et al., 2021). Curiously, despite the relatively “young” age of placental tissue (originating from the recent conception), the conceptus and placenta inherits maternal mitochondria—a major site where CPT1B is active. Thus, in certain respects, the placenta may share maternal age-related physiological characteristics.

In a human study, participants with lower skeletal muscle *CPT1B* mRNA expression were less able to oxidize lipids in a fasted state and were more insulin-resistant (Bétry et al., 2019). In mice, the age-associated decrease in skeletal muscle CPT1B protein exacerbated insulin resistance induced by a high fat diet, indicating that older mice had reduced metabolic flexibility in response to an obesogenic dietary challenge compared with their younger counterparts (Vieira-Lara et al., 2021). Meanwhile, loss of CPT1B activity in the heart increases myocardial lipids in obese mice and causes cardiac lipotoxicity in a heart failure mouse model (He et al., 2012; Zhang et al., 2016). These studies particularly highlight the importance of CPT1B in buffering metabolic stress and its contribution to overall metabolic health. As such, the age-related decline in placental CPT1B may similarly impair the placenta’s ability to appropriately regulate fatty acid oxidation in response to metabolic challenges such as maternal obesity; this could lead to dysregulated placental lipid metabolism and altered lipid-derived signaling, and ultimately placental dysfunction. Nonetheless, while our sample population encompassed a range of maternal BMI and glycemia, our study was restricted to those with relatively uncomplicated pregnancies with a livebirth following an elective cesarean section and thus not representative of the general obstetric population. Therefore, we were unable to test for associations of placental *CPT1B* expression with adverse pregnancy outcomes such as pre-eclampsia and stillbirth that are linked with placental dysfunction and advanced maternal age. Future studies in large cohorts that are adequately powered could be used to further investigate the link between placental *CPT1B* expression with these relatively infrequent adverse pregnancy outcomes.

### 4.3 Significance of the role of maternal BMI in influencing placental fatty oxidation

In addition to advanced maternal age, high BMI is another risk factor for stillbirth and antenatal complications such as GDM (Flenady et al., 2011; Plows et al., 2018). Studies of placentas from women with obesity generally report lower expression of CPTs, reduced acylcarnitines and impaired fatty acid oxidation, although the changes in expression of specific CPT isoforms differed between studies. For instance, Calabuig-Navarro et al. found that obesity increased placental *CPT2* mRNA expression, but decreased that of *CPT1B* and acylcarnitine content (Calabuig-Navarro et al., 2017), while Bucher et al. showed that CPT1A and CPT2 protein expression and acylcarnitines (16:0, 18:2, and 20:4) were reduced only in the placentas of female offspring (female placenta) among women with obesity (Bucher et al., 2021). In contrast, Powell et al. did not observe any changes with placental protein expression of CPTs, though they also demonstrated less fatty acid oxidation in female placentas of women with obesity (Powell et al., 2021). Similarly, we did not identify any associations of maternal BMI or infant sex with placental expression of CPTs. The discrepancies between studies may arise from different BMI cutoffs (i.e. overweight *versus* obese) and baseline population differences (e.g. Asian and non-Asian). Nonetheless, our finding of a maternal age-associated decline in placental acylcarnitines only among overweight women, provides additional supporting evidence that high BMI may contribute to reduced ability to process excess fatty acids.

Therefore, while maternal age is associated with decreased placental *CPT1B* expression, this only appears to impact acylcarnitine production when BMI is high, when the placenta is presumably already experiencing an increased fatty acid load. Hence, CPT1B may become the limiting factor in acylcarnitine production in an environment of excess fatty acids. Indeed, placental *CPT1B* mRNA expression positively associated with more acylcarnitines among overweight participants (BMI ≥25 kg/m^2^) as compared to just one significant association seen among the non-overweight participants (BMI <25 kg/m^2^), further highlighting the close relationship between maternal BMI, *CPT1B* expression and acylcarnitines in the placenta. Moreover, while placental fatty acid oxidation is reportedly reduced with GDM (Visiedo et al., 2013), GDM status had minor implications on the associations of placental *CPT1B* expression and of maternal age with placental acylcarnitines in our cohort, which suggests that differences in BMI are more important than differences in maternal glycemia.

### 4.4 Role of other placental CPTs

In addition to being the only CPT associated with age, *CPT1B* mRNA expression was positively associated with the largest number of placental acylcarnitines, suggesting it may be the main transferase for converting medium and long-chain fatty acids into acylcarnitines in the human placenta. This is similar to a previous finding showing that placental *CPT1B* mRNA expression positively correlated with total placental acylcarnitine content (Calabuig-Navarro et al., 2017). The positive relationships of placental *CPT1C* mRNA expression with only the longer chain acylcarnitines (16:0, 18:0 and 18:2) suggests its particular importance in generating the very long chain acylcarnitines. This is corroborated by the localization of CPT1C mainly in the endoplasmic reticulum, hinting at its role in biosynthesis as opposed to catabolism (in mitochondria) and that the loss of CPT1C results in decreased long chain signaling endocannabinoid production (Sierra et al., 2008; Lee and Wolfgang, 2012). Unexpectedly, placental *CPT1A* expression was negatively associated with acylcarnitines. The underlying reasons remain unclear, but one possibility is that placental increases in CPT1A enhances fatty acid oxidation overall, such that longer chain acylcarnitines become depleted. This is similar to the negative relationship seen in patients with chronic kidney disease, where decreased kidney *CPT1A* mRNA expression was linked with increased accumulation of short and middle chain acylcarnitines (Miguel et al., 2021). In contrast, CPT2 was not associated with any placental acylcarnitine in our cohort, which suggests it is not the limiting factor in the placenta for synthesis of the medium and long-chain acylcarnitines examined. Nonetheless, as there are no inhibitors currently available to selectively block the activity of each CPT in isolation, we are limited in our ability to determine the specific role of each CPT in *vitro* studies of the human placenta.

### 4.5 Possible mechanisms for CPT1B decline with age and potential reversal with carnitine supplementation

The mechanisms by which maternal age affects placental *CPT1B* expression are unknown. However, insights may be gained from non-placental studies. For example, decreased CPT1 expression with increasing age in tissues such as the heart and skeletal muscle is speculated to result from cumulative mitochondrial oxidative damage over time (Odiet et al., 1995; Vieira-Lara et al., 2021). As such, oxidative damage accumulated in the maternal mitochondria of the aging oocyte that are subsequently inherited by the fetus may be one contributing factor. *In vitro* studies conducted on placental explants show that acute oxidative stress of up to 4 h does not affect placental CPT1B expression at the mRNA or protein level (Thomas et al., 2019), but the effects of chronic oxidative stress remain to be investigated. Direct signaling from the maternal tissues to the placenta may also influence placental CPT expression, given that advanced maternal age can impair decidualisation and thus alter the biochemical and hormonal environment that the developing placenta is exposed to (Woods et al., 2017; Mendes et al., 2020). Alternatively, a decline in CPT may be due to deficiency of micronutrients needed for optimal fatty acid oxidation. For instance, in conjunction with the age-associated drop in CPT (Bétry et al., 2019; Vieira-Lara et al., 2021), skeletal muscle carnitine content also decreases with age in humans and mice (Costell et al., 1989). Interestingly, carnitine supplementation was able to enhance CPT1 transcription in the liver of aged rats (Karlic et al., 2002). In humans, pregnancy also results in a decline in circulating carnitine (Keller et al., 2009), and dietary carnitine supplementation can increase hepatic CPT1B activity in pregnant pigs (Xi et al., 2008). Furthermore, carnitine supplementation was previously shown to decrease the stillbirth rate in sows (Eder, 2009). Exercise can also increase skeletal muscle and adipose tissue *CPT1B* mRNA expression in young and middle adults across the BMI spectrum (Lohninger et al., 2005; Otero-Díaz et al., 2018) but whether exercise in pregnancy can increase placental CPT1B remains to be investigated. The notion that maternal physical activity can influence fetal-placental tissues at a molecular level was suggested in a study by Chaves et al. (Chaves et al., 2022), which demonstrated that maternal exercise altered metabolism in isolated umbilical cord mesenchymal stromal cells. It is thus tantalizing to speculate that the CPT-promoting effects of carnitine supplementation and exercise individually or in combination may be able to counter the age-associated placental CPT decline in pregnancy and possibly reduce advanced maternal age-linked stillbirths and other pregnancy adversity, which could be explored in future studies. Therefore, further studies are warranted to improve understanding of CPT regulation at the maternal-fetal interface.

## 5 Conclusion

In summary, older maternal age is specifically associated with lower placental *CPT1B* expression and *CPT1B* appears to be the main CPT that catalyzes acylcarnitine production in the placenta. However, placental acylcarnitines are only lower with older maternal age in overweight/obese women. These findings may underlie decreased placental metabolic flexibility and ability to adapt to adverse intrauterine environments, which may contribute to greater risk of pregnancy complications in older women, particularly if they are overweight/obese.

## Data Availability

The raw data supporting the conclusion of this article will be made available by the authors upon reasonable request.
