# Supplementary material for: Increasing maternal age associates with lower placental CPT1B mRNA expression and acylcarnitines, particularly in overweight women
Source: Front Physiol. 2023 May 18;14:1166827. doi: 10.3389/fphys.2023.1166827 (PMC10232777; doi:10.3389/fphys.2023.1166827)

Supplementary information

Supplementary methods

Chromatography was performed using a 2.1 x 100 mm 1.8 µm Zorbax Eclipse Plus C18 RRHD (Agilent Technologies) column at 60°C, and the following gradients and settings: Mobile phase A: 50% water, 30% acetonitrile, 20% isopropanol, 10 mmol/L ammonium formate and Mobile phase B: 90% isopropanol, 9% acetonitrile, 1% water, 10 mmol/L ammonium formate; Start (0.4 ml/min): 90% A, 0-2.7minutes: decrease to 55% A, 2.7-2.8 minutes: decrease to 47% A, 2.8-9 minutes: decrease to 35% A, 9-9.1 minutes: decrease to 11% A, 9.1-11 minutes: decrease to 8% A, 11-11.1 minutes: decrease to 0% A, 11.1-11.9 minutes: 0% A, 11.9-12 minutes: Increase to 90% A, 12-15 minutes: 90% A. The Agilent 6490 triple quadrupole was run with the following settings - Gas temperature: 150 °C; gas flow: 17 L/min; nebulizer: 20 psi; sheath gas temperature: 200°C; sheath gas flow: 10 L/min; positive capillary voltage: 3500 V; negative capillary voltage: 3000 V; positive nozzle voltage: 1000 V; negative nozzle voltage: 1500 V; positive high pressure RF (iFunnel): 100 V; negative high pressure RF (iFunnel): 90 V; positive low pressure RF (iFunnel): 100 V; negative low pressure RF (iFunnel): 60 V; fragmentor: 380; polarity: positive. Acylcarnitines were measured using the transitions shown in Supplementary table 1.

Supplementary Table 1. Transitions used for the measurement of placental acylcarnitines.

Name	Retention time (min)	Collision Energy	Precursor Ion (m/z)	Product Ion (m/z)
AcylCarnitine 12:0	1.3	30	344.3	85.1
AcylCarnitine 13:0	1.7	30	358.3	85.1
AcylCarnitine 14:0	2.0	30	372.3	85.1
AcylCarnitine 14:1	1.5	30	370.3	85.1
AcylCarnitine 14:2	1.2	30	368.3	85.1
AcylCarnitine 15:0	2.3	30	386.3	85.1
AcylCarnitine 16:0	2.5	30	400.4	85.1
AcylCarnitine 16:0 d3 (Internal standard)	2.5	30	403.3	85.1
AcylCarnitine 16:1	2.2	30	398.3	85.1
AcylCarnitine 17:0	2.9	30	414.4	85.1
AcylCarnitine 18:0	3.6	30	428.4	85.1
AcylCarnitine 18:1	2.7	30	426.4	85.1
AcylCarnitine 18:2	2.2	30	424.3	85.1
AcylCarnitine 20:4	2.3	30	448.4	85.1
AcylCarnitine 22:6	3.6	30	472.4	85.1

Supplementary Figure 1. Associations between abundance of placental acylcarnitines with placental *CPT1B* mRNA expression or maternal age by linear regression stratified by maternal GDM status. The forest plots show coefficient estimates and 95% confidence intervals of associations between placental acylcarnitines (outcome) with placental *CPT1B* mRNA expression (A) or maternal age (years, B) in subjects with normoglycemia (n=26) and GDM (n=24). Filled symbols show acylcarnitines that are significantly associated after adjustment by Benjamini-Hochberg's correction. Data for placental acylcarnitine abundance and mRNA expression data of *CPT1B* were log$_2$-transformed then converted to Z-scores prior to linear regression.

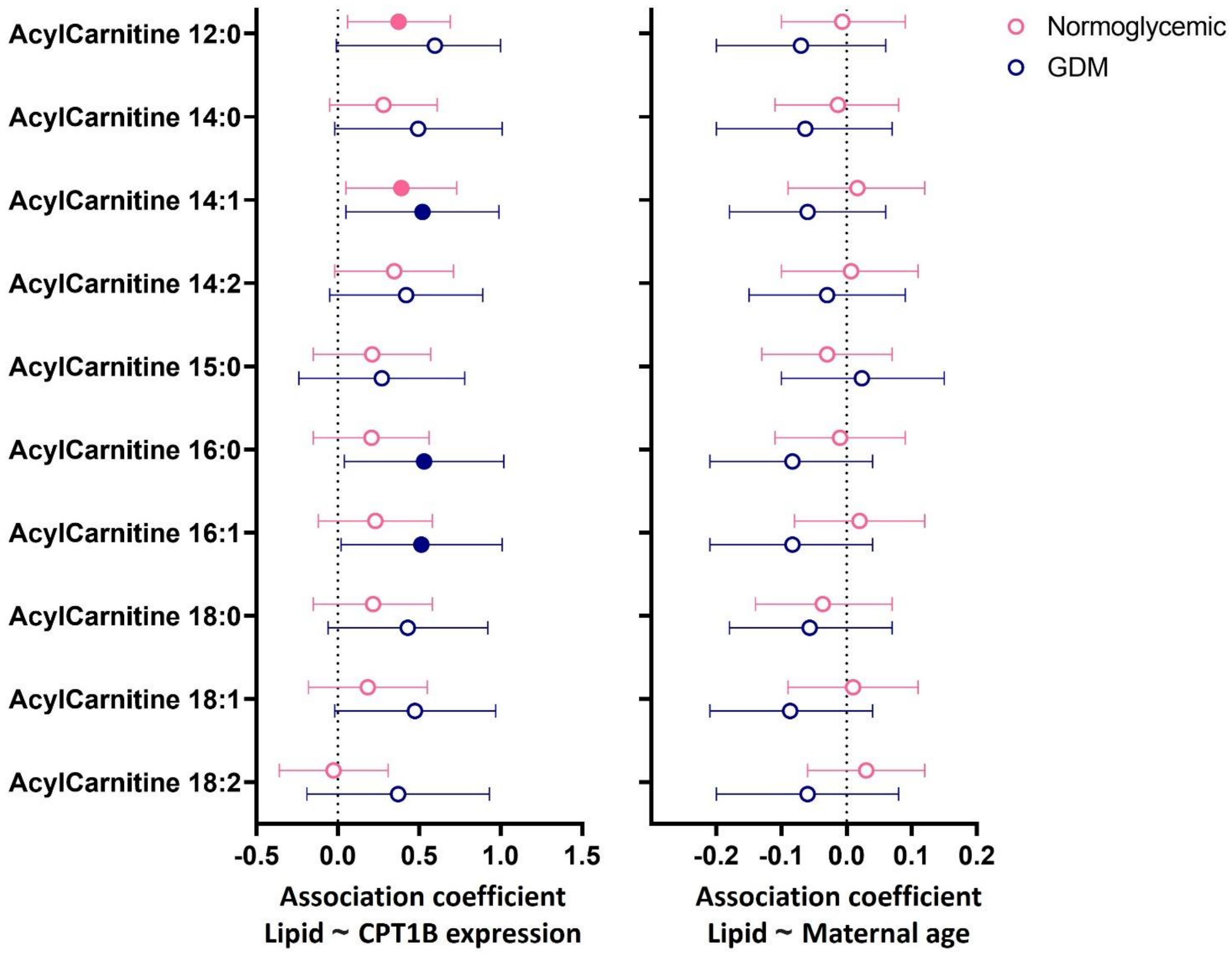

Supplement: Supplementary file 1 [file Presentation1.pdf]
